# Supplementary figures and images for: Speech-in-noise discriminability after noise exposure: Insights from a gerbil model of acoustic trauma
Source: PLoS One. 2025 Sep 22;20(9):e0330663. doi: 10.1371/journal.pone.0330663 (PMC12453242; doi:10.1371/journal.pone.0330663)

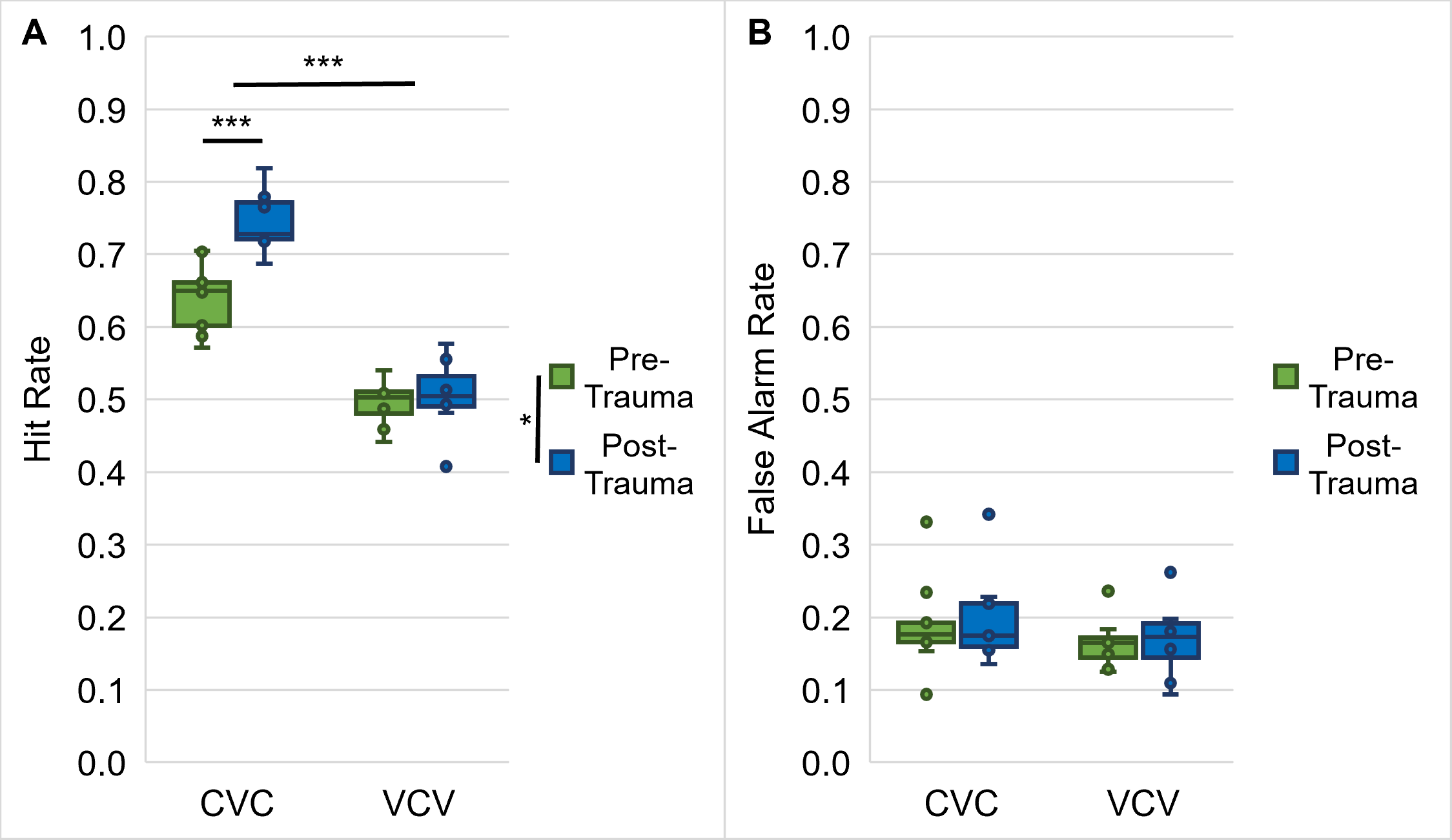

Supplement: S1 Fig — Hit rates (A) for the behavioral performance of the gerbils (n = 9) were significantly higher for CVC conditions (but not for VCV conditions) post-trauma compared to pre-trauma. Neither the noise trauma nor the logatome type had an effect on the false alarm rate (B). *: p < 0.05, ***: p < 0.001. (TIF) [file pone.0330663.s001.tif]
